# Exploring demographic, healthcare, and socio-economic factors as predictors of COVID-19 incidence rate: A spatial regression analysis

**DOI:** 10.1371/journal.pone.0312717

**Published:** 2024-10-28

**Authors:** Kittipong Sornlorm, Ei Sandar U, Wongsa Laohasiriwong, Wor Mi Thi

**Affiliations:** Faculty of Public Health, Khon Kaen University, Nai Mueang, Mueang Khon Kaen, Khon Kaen, Thailand; Al Mansour University College-Baghdad-Iraq, IRAQ

## Abstract

This study investigated the relationship between demographic, healthcare, and socio-economic factors, and COVID-19 incidence rate per 100,000 population in Thailand at the province level between January 2020 and March 2022, using a five-phase approach by spatial analysis. OLS models were initially used with significant variables: household, hospital, and industry density, nighttime light index (NTLI). Spatial dependency led to spatial error (SEM) and spatial lag models (SLM), performing better with similar significant variables being applied. SEM explains 58, 65 and, 70 percent in Wave 1, 4 and 5 of COVID-19 variation. SLM explains 25 and 76 percent in Wave 2 and 3 of incidence rate. Positive associations were found between incidence and household density, hospital/medical establishments with beds, Nighttime Light Index (NTLI), and negative with population, hospital, and industry density. Wave 5 showed significant changes with negative for household, hospital, and industry density, urban population; positive for hospital/medical establishments with beds, internet access, NTLI. The study showed that significant predictors of COVID-19 incidence rate vary across waves. Population, household and hospital density, urbanization, access to medical facilities, industrialization, internet access, and NTLI all play a role. The study suggests SEM and SLM models are more appropriate, providing useful information for policymakers and health officials in managing pandemic in Thailand.

## 1. Introduction

As of March 15, 2022, the COVID-19 pandemic had a significant effect worldwide with over 512 million confirmed cases and 6 million deaths reported to the World Health Organization (WHO) [1]. The emergence of the COVID-19 pandemic can be traced back to late 2019 in Wuhan, China. On March 11, 2020, WHO declared it a pandemic [2]. In early January 2020, Thailand reported its first case of COVID-19 [3], and since then, multiple waves of COVID-19 have been reported in the country, with a total of 4,281,536 cases and 28,778 deaths reported as of May 5, 2022 [4].

Studies have explored various aspects of COVID-19, including its spread, incubation period, symptoms, and effects on different populations. Sannigrahi et al. found that income, poverty, and population had a weighty impact on COVID-19 associated deaths in European countries [5]. Miller et al. identified Spain, Italy, China, and Iran as the most affected countries in March 2020 [6]. Kianfar and Mesgari found that poverty and elderly populations are important factors in the incidence rate of COVID-19 and that they are more vulnerable to severe illness and death [7]. Luenam et al. found that during both major pandemic waves, there was a significant negative association between the COVID-19 incidence rate and nighttime light index in Thailand [8]. However, further research is needed to better recognize the impact and level of association of various factors on the incidence rate in Thailand and to help authorities make informed decisions [9].

Thailand’s unique geography, population, and socio-economic characteristics make it a valuable location for studying the impact of various factors on the incidence of infectious diseases. This study aimed to achieve the following objectives: to identify high-risk areas for the spread of COVID-19 in Thailand using spatial regression analysis with Geographic Information Systems (GIS), and to explore the relationship between demographic, healthcare, and socio-economic factors and the incidence of COVID-19 at the provincial level. By identifying high-risk areas, our study can guide targeted interventions such as vaccination campaigns and improved disease surveillance systems to reduce transmission risk. By using spatial regression analysis to explore the relationship between demographic, healthcare, and socio-economic variables at the province level, this study has provided important insights into the key points for interventions to curb the spread of the pandemic.

## 2. Methods

### 2.1 Data collection and preparation

The study analyzed data for the entire COVID-19 period in 77 provinces of Thailand, from January 2020 to March 2022, which was divided into five phases based on the number of cases and waves [10]. The dependent variable was “COVID-19 cases per 100,000 population” was the id and a total of 2,569,617 confirmed cases were analyzed for a period of 27 months. The study period covered the entire pandemic phase in Thailand and was divided into five phases: “First wave (Jan-May 2020), Second wave (Sep 2020-Mar 2021), Third wave (Apr-Jun 2021), Fourth wave (Jul-Oct 2021), and Fifth wave (Nov 2021-Mar 2022)” [4].

The study’s independent variables included demographic, healthcare, and socio-economic factors. Demographic data on population and household density and urbanization were obtained from “National Statistical Office of Thailand”. Healthcare data on hospital density, medical establishments with beds which was measured with number of hospital beds per 10,000 population, physician and volunteer healthcare workers, and primary healthcare centers were obtained from the “Ministry of Public Health”. Socio-economic statistics on industry density, monthly household income, internet access, and nighttime light index were retrieved from various sources, including the “Department of Industrial Works and the Ministry of Digital Economy and Society” [11–13].

### 2.2 Statistical methods

In this study, the retrospective data on incidence rate and demographic, healthcare, and socio-economic factors were used to compile a dataset at the province level for analysis. This data was joined to the boundary shapefiles of the provinces using QGIS software [14]. The study used GeoDa version 1.18.0.16 to perform spatial autocorrelation analysis in the spatial regression analysis [15]. For univariate and bivariate analysis, the study used 3 k-Nearest Neighbor provinces as the spatial weight matrix.

## 2.2.1 Moran’s I and local Moran’s I

Geospatial statistics such as global and local Moran’s I and the Local Indicator of Spatial Association (LISA) were used to conduct autocorrelation studies [14]. Moran’s I is a statistical tool used to find out the association between variables in a spatial context. The values range from -1 to 1, where +1 with strong positive spatial autocorrelation and -1 with negative spatial autocorrelation [15–17]. The study used 999 permutation simulations at a significance level of p = 0.05 to assess spatial autocorrelation reliably.

The “Moran’s *I* test” is calculated as follows:

$$I=\frac{\Sigma_{i}\Sigma_{j}w_{ij}z_{i}{.z}_{j}/S_{0}}{\Sigma_{i}z_{i}^{2}/n}$$ (Eq 1)

Where *Σ*_*i*_ is “the sum of variable of interest at the location *I”*; *Σ*_*j*_ is “the sum of variable of interest at the location *j*; *w*_*ij*_ is “the elements of the spatial weight matrix”; *z*_*i*_ is the term $i-\bar{x}$ (where $\bar{x}$ expresses “the mean of variable *x* for an observation at location *i"*); *z*_*j*_ is the term $x_{j}-\bar{x}$ (where $\bar{x}$ expresses “the mean of variable *x* for an observation at location *j”*); S_0_ “the sum of all weights Σ_i_Σ_j_w_ij_”; and *n* is “the number of observations”.

Inability to find the local spatial autocorrelation is the limitation of Global Moran’s I. To overcome this, the equation was Anselin (2020) extended this statistic to a version offering the specific locations of spatial autocorrelation [16], which is calculated as follows:

$$I_{i}=c.z_{i}\sum_{j}w_{ij}z_{j}$$ (Eq 2)

where Σ_j_ is “summation of all variables of interest at location *j* that borders location *I”*; *z*_*i*_ is “the deviation of the variable of interest from its mean value at location *i"*; *z*_*j*_ is “the deviation of the variable of interest from its mean value at location *j”*; *w*_*ij*_ is “the elements of the spatial weight matrix”; and c is “a constant that is typically set to 1/n”.

For developing the spatial model, Queen’s contiguity 1 was used as the weight matrix. The study used three different models for spatial regression—Ordinary Least Squares (OLS), Spatial Lag Model (SLM), and Spatial Error Model (SEM)—to explore the relationship between potential explanatory variables and the COVID-19 incidence rate.

## 2.2.2 Spatial Lag Model (SLM)

The SLM is a regression analysis method that includes a spatial lag of the dependent variable as a predictor to account for spatial dependence, with the spatial lag being calculated as the weighted average of the dependent variable in neighboring areas. The SLM can be expressed as:

Y=α+ρWy+Xβ+ε (Eq 3)

where:

α = the intercept term,

Y = dependent variable

X = matrix of independent variables

β = vector of coefficients to be estimated

ε = error term

W = spatial weights matrix

ρ = spatial autoregressive parameter

Therefore, the Spatial Lag Model (SLM) captures the spillover effect of the dependent variable on bordering areas, and the direction of spatial dependence, while also identifying the association between spatial dependence and independent variables [17].

## 2.3.3 Spatial Error Model (SEM)

The SEM is another type of regression analysis that accounts for the spatial dependence among the error terms [18]. The Spatial Error Model is expressed in Equation:

Y=α+Xβ+u,u=λWu+ε (Eq 4)

where

**α** = the intercept term,

***β*** = the regression coefficient,

X = explanatory regression variable,

*ε* = the error term vector,

**λ** = the spatial error coefficient

W = the spatial weight matrix.

The SEM, unlike the SLM, accounts for the spatial dependence in the error terms and captures the relationship between the spatial dependence in the error terms and the independent variables, enabling the identification of spatial patterns in the residuals and the direction of the spatial dependence [17].

In the SLM, the spatial dependence is netted by including a spatial lag of the dependent variable as a predictor, while it is captured by the error term, which is spatially correlated in the SEM. Both models are estimated using the maximum likelihood method, which considers the spatial dependence present in the data [19], making them useful for analyzing geographical data such as relationships among the independent and COVID-19 incidence rate in Thailand, helping to inform effective policy and control measures for the pandemic.

## 2.2.4 Procedure of analysis

The goal of using Moran’s I was to identify the spatial patterns of demographic, healthcare, and socioeconomic factors concerning with COVID-19 incidence rate. By performing univariate and bivariate analysis, the researchers identified which variables were significant in explaining the incidence rate and used this formula to determine if the distribution of the disease was clustered, dispersed, or random. When this value is positive, it indicates that these are clustered, meaning that similar values tend to be close to each other. Conversely, a negative value indicates that the values are dispersed, meaning that dissimilar values are found near each other. A value of zero means that there is no spatial autocorrelation, and the variable are randomly distributed with respect to location [15].

The study used stepwise forward selection to choose explanatory variables for an OLS model and checked for multicollinearity using VIF. However, if significant spatial autocorrelation was detected, OLS could not be used. In such a scenario, the study chose the model with the largest test value among the robust models. The selected model was then based on its best R^2^, log-likelihood values, and a low Akaike information criterion (AIC) score. This process was done to identify the most suitable model for analyzing the COVID-19 infection rate among the population in Thailand and to minimize any biased results due to spatial dependence [17].

## 2.2.5 Ethical considerations

The study received approval from the “Ethics Committee in Human Research of Khon Kaen University in Thailand”. The reference number "HE652161" is provided for further reference or verification.

## 3. Results

### 3.1 Moran’s I among COVID-19 incidence rate in five phases of pandemics

Local Moran’s I determined the spatial autocorrelation among COVID-19 incidence rate in five phases of pandemics. In five phases, Local Moran’s I were 0.145 (p = 0.051), 0.043 (p = 0.040), 0.622 (p = 0.001), 0.535 (p = 0.001), and 0.454 (p = 0.001), as shown in Table 1. The COVID-19 incidence rate is not evenly distributed among the provinces and there is a relationship between the incidence rate in one province and its neighboring provinces. The spatial autocorrelation indicates that COVID-19 incidence rate tends to cluster in some regions and the likelihood of having a high incidence rate in a province is higher if its neighboring provinces also have a high incidence rate (Table 1).

**Table 1:** Univariate analysis of COVID-19 incidence rate in five phases and factors of Interest (permutation: 999).

Variables	Moran’s I
**Phases of COVID_19**	
Phase of Wave 1	0.145
Phase of Wave 2	0.043*
Phase of Wave 3	0.622**
Phase of Wave 4	0.535**
Phase of Wave 5	0.454**

Footnote 1.

* *P* <0.05

** *P* <0.001

### 3.2 Moran’s I of explanatory variables with COVID-19 incidence rate in five phases of pandemics

**Fig 1:**
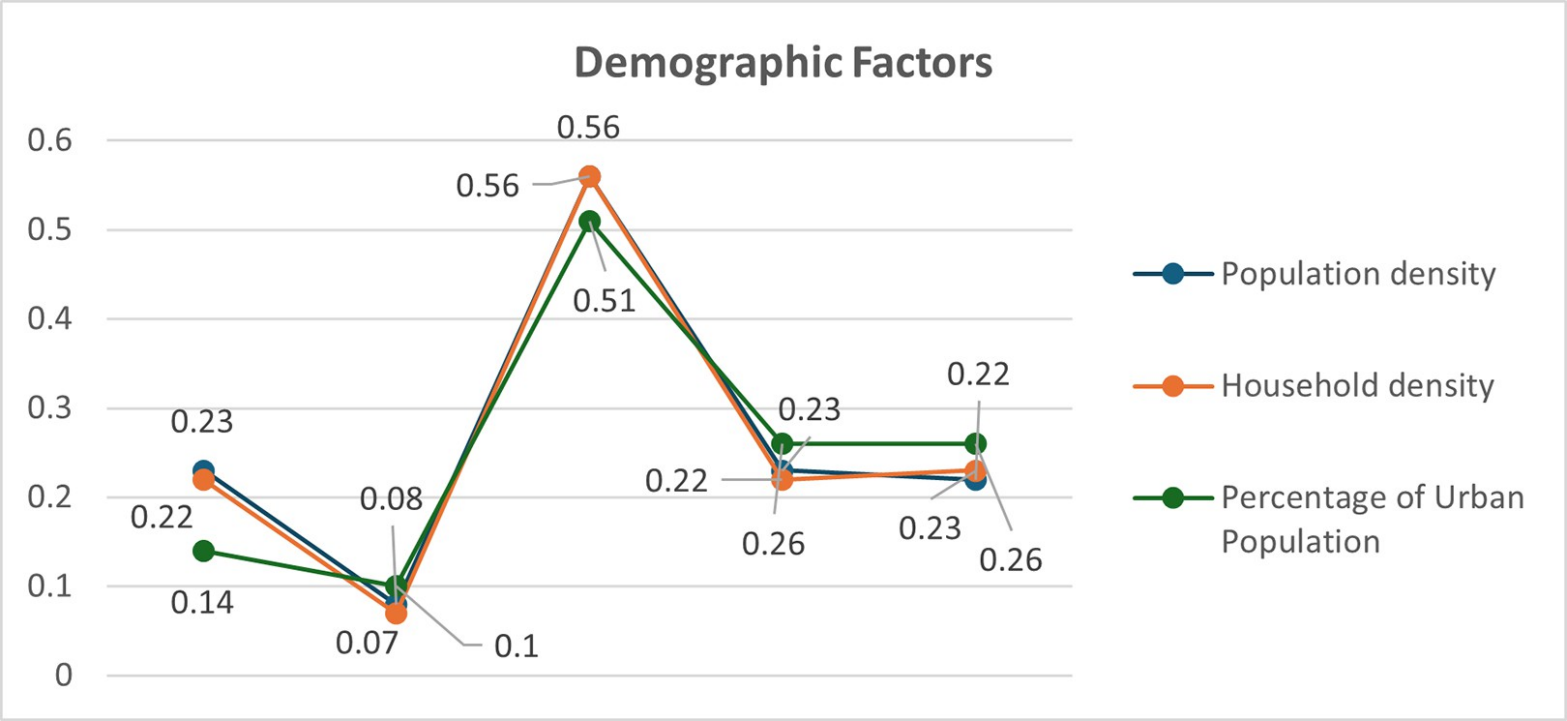
Distribution of local Moran’ I by bivariate analysis of demographic factors with COVID-19 incidence rate (permutation: 999).

**Fig 2:**
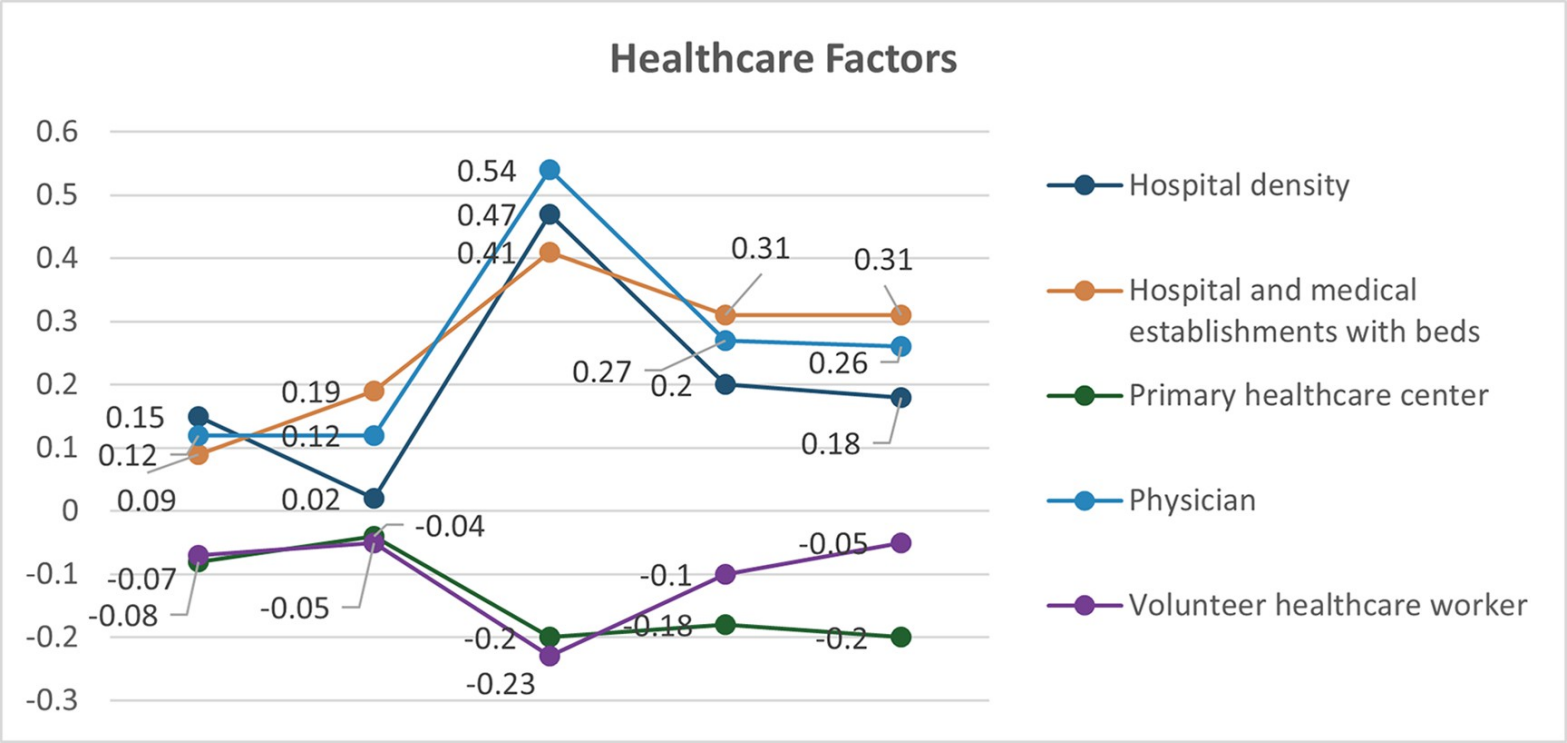
Distribution of local Moran’ I by bivariate analysis of healthcare factors with COVID-19 incidence rate (permutation: 999).

**Fig 3:**
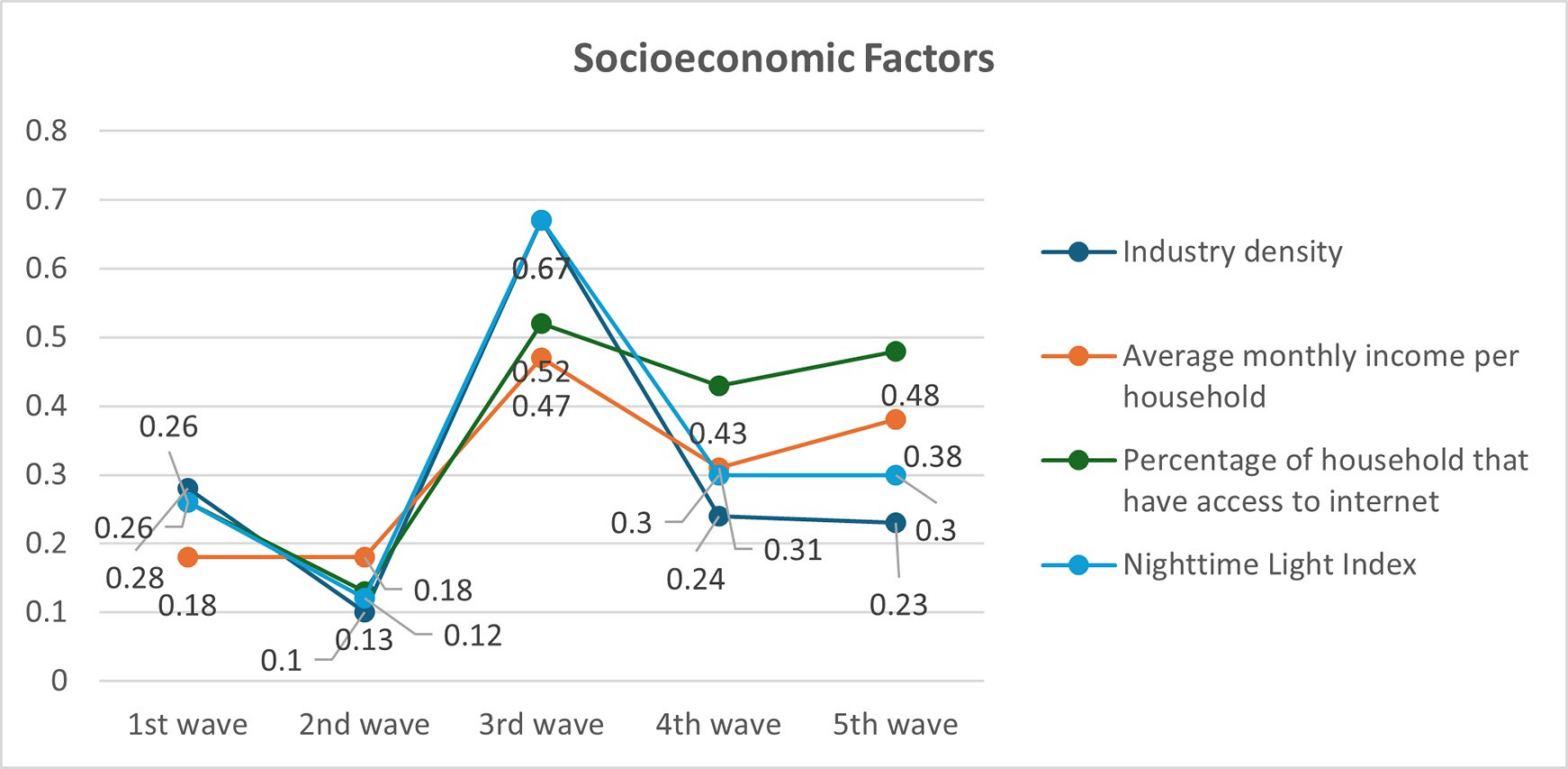
Distribution of Local Moran’ I by bivariate analysis of socio-economic factors with COVID-19 incidence rate (permutation: 999).

The bivariate analysis of explanatory variables and COVID-19 incidence rate revealed significant spatial associations through the Moran’s I test. Moran’s I values range from -1 to 1, where positive values indicate positive spatial autocorrelation, negative values indicate negative spatial autocorrelation, and values near zero suggest no spatial autocorrelation. All examined variables demonstrated statistically significant spatial associations (p<0.05) with COVID-19 incidence rates across all pandemic waves, indicating that the spatial distribution of these factors was related to the spatial patterns of COVID-19 incidence in Thailand. Population density, household density, urban population, hospital density, medical facilities, industry density and average household income, internet access, and nighttime light index were consistent with the spatial correlation pattern of COVID-19 cases. Household density exhibited the strongest positive spatial association (Moran’s I > 0.5 across all waves), followed by urban population percentage. Population density showed a weaker but consistent positive association (Fig 1). Hospital and medical establishments with beds showed the strongest positive spatial association, particularly in waves 3–5 (Moran’s I > 0.4). Hospital density and primary healthcare centers demonstrated weaker associations, with some fluctuations across waves (Fig 2). The nighttime light index and industry density consistently showed strong positive spatial associations (Moran’s I > 0.5 for most waves). Internet access and average monthly income exhibited moderate positive associations that strengthened in later waves (Fig 3). These results suggest that areas with higher values of these factors tend to be surrounded by areas with higher COVID-19 incidence rates. The varying strengths of associations across waves indicate the dynamic nature of the pandemic’s spatial patterns and the changing influence of different factors over time.

### 3.3 Spatial regression of explanatory variables with COVID-19 incidence rate in five phases of pandemics

The significant variables for the COVID-19 incidence rate in Thailand have been identified in each wave accoding to OLS model (Table 2); household, hospital and industry density per km^2^ and nighttime light index in Wave 1; population and household density per km^2^, and nighttime light index in Wave 2; household density per km^2^ and hospital and medical establishments with beds in Wave 3, population and household density per km^2^, and nighttime light index in Wave 4; household, hospital and industry density per km^2^, percentage of urban population, hospital and medical establishments with beds, access to internet, and nighttime light index in Wave 5. The OLS explains approximately 52% (R^2^ = 0.52) in wave 1, 25% (R^2^ = 0.25) in wave 2, 72% (R^2^ = 0.72) in wave 3, 35% (R^2^ = 0.35) in wave 4, and 70% (R^2^ = 0.70) in wave 5 of the variation in the incidence rate in Thailand.

**Table 2:** Spatial regression models summary with factors influencing COVID-19 incidence rate in Wave 1 to 5 of Thailand.

	WAVE-1		WAVE-2		WAVE-3		WAVE-4		WAVE-5	
	SLM	SEM	SLM	SEM	SLM	SEM	SLM	SEM	SLM	SEM
Constant	0.11	**-0.07**	**22.47** **	25.29 **	**-106.38** **	-89.12	627.39 **	**1844.03** ***	-2042.23 **	**-1817.84** *
Density of population per km^2^	NS	NS	**-0.42** ***	-0.46 ***	NS	NS	-6.57 **	**-9.20** ***	NS	NS
Density of household per km^2^	-0.03 ***	**-0.03** ***	**0.73** ***	0.78 ***	**0.56** ***	0.65 ***	8.34	**13.59** ***	-9.35 ***	**-9.20** ***
Percentage of urban population	NS	NS	NS	NS	NS	NS	NS	NS	-24.98 **	**-22.66** *
Density of hospital per km^2^	-0.003 **	**-0.002** *	NS	NS	NS	NS	NS	NS	-0.42 *	**-0.37** *
Hospital and Medical Establishments with Beds	NS	NS	NS	NS	**6.68** **	6.87 **	NS	NS	32.75 *	**32.36** *
Density of Industry per km^2^	-9.37 **	**-10.34** ***	NS	NS	NS	NS	NS	NS	-1018.46 *	**-1025.77** *
Access to internet	NS	NS	NS	NS	NS	NS	NS	NS	46.16 ***	**42.38** ***
Nighttime Light Index	4.36 ***	**4.81** ***	**12.25** *	13.02	NS	NS	483.08 ***	**503.75** ***	983.25 *	**976.08** ***
**ρ**	0.66		**0.07**		**0.42**		0.63		0.66	
**λ**		**0.45**		0.18		0.32		**0.70**		**0.14**
R–squared	0.53	**0.58**	**0.25**	0.26	**0.76**	0.74	0.63	**0.65**	0.70	**0.70**
Log likelihood	-219.79	**-216.61**	**-405.33**	-405.01	**-482.88**	-487.1	-647.36	**-646.45**	-699.63	**-611.15**
AIC	451.58	**443.22**	**818.65**	818.01	**973.76**	982.2	1304.71	**1300.90**	1240.40	**1238.30**
BIC	465.65	**454.94**	**828.37**	828.38	**983.13**	991.58	1316.43	**1310.28**	1261.49	**1257.05**
Lagrange Multiplier	p = 0.50	**p = 0.02**	**p = 0.07**	p = 0.45	**p<0.001**	p = 0.18	p<0.001	**p<0.001**	p = 0.06	**p = 0.012**

Footnote 2

* denotes P<0.05

** denotes P <0.01

*** denotes P <0.001

Footnote 3: Variables are listed with their coefficients and significance levels. "NS- Not significant" indicates that the variable was included in the model but did not reach statistical significance.

The performance of OLS, SEM, and SLM models was compared using R-squared, AIC, and log-likelihood values (Table 2). The spatial models outperformed the OLS model across all waves. For Waves 1, 4, and 5, the SEM showed improvements in R-squared values (0.58, 0.65, and 0.70 respectively) and reductions in AIC compared to OLS. For Waves 2 and 3, the SLM demonstrated better performance, with R-squared values of 0.25 and 0.76 respectively. The Lagrange multiplier test indicated spatial dependence in the error term for Waves 1, 4, and 5, and spatial dependence in neighboring places for Waves 2 and 3. Based on these results, SEM was selected as the most appropriate model for Waves 1, 4, and 5, while SLM was chosen for Waves 2 and 3. These spatial models reflect the spatial dependence in the data and provide a more accurate representation of the relationships between the variables and COVID-19 incidence rates across the different waves of the pandemic in Thailand.

The explanatory variables that remained significant over the waves in their relationships with the COVID-19 incidence rate in Thailand were household density which showed significance across all five waves, indicating its persistent influence on the incidence rate of COVID-19 throughout the study period; nighttime light index remained significant in Waves 1, 2, 4, and 5, indicating its continued relevance in the analysis over multiple waves. Some variables remain significant on the respective waves; hospital and industry density per km^2^ in Wave 1: population density per km^2^ in Wave 2: hospital and medical establishment with beds in Wave 3:, population density per km^2^ in Wave 4, and percentage of urban population hospital density per km^2^, hospital and medical establishment with beds, industry density per km^2^ and access to internet in Wave 5. A positive relationship was found between the incidence rate and household density in Waves 2 to 4, hospital and medical establishments with beds in Wave 3, and nighttime light index in Waves 1, 2, and 4. However, there was an inverse relationship between the incidence rate and population density in Wave 2 and 4, hospital density in Wave 1, and industry density in Wave 1. In Wave 5, the association between the factors and incidence rate changed significantly compared to previous waves, with a negative relationship between the incidence rate and household density, urban population, hospital density, and industry density, and a positive relationship between the incidence rate and hospital and medical establishments with beds, access to the internet, and nighttime light index (Table 2).

## 4. Discussion

This study examined the spread of COVID-19 in Thailand from January 2020 to March 2022, with a focus on exploring its spatial patterns and the changing relationships between demographic, healthcare, and socioeconomic factors, and the spread of pandemic. The study aimed to provide information that could inform public health policies at the provincial and national level on the spatial transmission of COVID-19. Despite over two years of the pandemic, many countries are still facing significant outbreaks and the occurrence of new variants presents additional challenges for pandemic control.

In Thailand, the pattern of the pandemic shifted over time, as evidenced by the clustering of cases in waves 3 to 5. During all five phases, there was a significant positive spatial autocorrelation of COVID-19 incidence, meaning areas with high cases were likely surrounded by others with high cases. The study found that the spread of diseases in Thailand mainly alternated between random and clustered patterns over time, potentially due to the intervention measures establishment and regulated at different phases [20]. The study emphasized the importance of early control of cases to prevent widespread spread. Additionally, the study observed different spatial hotspots at different times, suggesting a potential trend of the virus spreading from central areas to other areas.

The highest autocorrelation degree of cases during lockdown period was observed in Wave 3 with COVID-19 Delta variants. This finding was contrary to the expectation of a reduction in spatial autocorrelation during lockdown period. In Thailand, as of December 23, 2022, 77% of the population received a full vaccine coverage and 82% received at least one dose, following the initiation of the vaccination campaign during the third wave [21]. Although low vaccination coverage and irrational health behaviors were significant concerns, their impact was indeed notable in earlier waves. (Waves 1 and 2). The unexpected increase in spatial autocorrelation can be attributed to several additional factors in Wave 3. The Delta variant’s increased transmissibility and virulence contributed significantly to the clustering of cases. Moreover, the nature of the pandemic during Wave 3 led new patterns of transmission and health responses that may not have been fully accounted for in the previous waves. These factors might play a crucial role in the observed spatial autocorrelation. However, the gradual decrease in the degree of spatial autocorrelation in waves 4 and 5 compared to previous waves implies the effectiveness of measures such as isolation, detection, and vaccination in mitigating the spread of the virus. One study from Singapore observed the similar spatial patterns of the spread in Singapore [22].

This analysis shows that demographic, healthcare, and socioeconomic factors play a weighty position in shaping the spread over the provinces of Thailand. The important factors varied in their impact during changed phases of the pandemic. The density of households was a crucial factor affecting transmission rates over all waves. The nighttime light index, as a proxy for economic activity and movement, varied in its impact across different waves.

During the Wave 1, the alpha variant of the virus and the government’s COVID-19 control policies might have been effective in mitigating the spread of the virus. Moreover, these variables might play a crucial role in slowing the spread, as evidenced by inverse relationships with household density, industry density, and hospital density [23]. These factors played a crucial role in slowing the spread initially. However, the incidence rate increased within both the province and surrounding provinces that shared similar nighttime light indices, showing that areas with higher economic activity and movement experienced continued spread.

From Waves 2 to 4, the variant of the virus may have more virulence and transmissible (especially in Wave 3 and 4) that might have contributed to higher incidence rates. Additionally the control measures may have weakened in some areas, especially in those with high household density, high numbers of hospital and medical establishments, and high nighttime light areas. However, the hospital density was negatively correlated with incidence rate in Wave 1 and Wave 5. This implied that hospital density can have a significant impact as a protective factor in certain phases of the COVID-19 pandemic, but in times of rapid spread and high numbers of infected individuals, medical facilities especially hospital and medical establishment with beds may become overwhelmed and insufficient as observed in Wave 3 and Wave 5). This overwhelmed capacity contributed to the challenges in managing the outbreak effectively [22, 24].

In the 5^th^ wave (Omicron wave), an inverse relationship between certain factors and incidence rate, including household density, densely populated urban areas, and hospital and industry density were observed. However, factors such as access to medical facilities, internet access, and the nighttime light index showed a positive association with the incidence rate. This suggests that government control measures were stronger in some areas but still challenging in economically and socially accessible areas. Furthermore, the increased demand for medical facilities, especially hospitals and medical establishments with beds, may have led to the potential for these resources to become overwhelmed and insufficient. These findings highlight the ongoing challenges in controlling the spread of diseases and the significance of considering multiple factors in developing effective strategies to control the pandemic.

Population density and household density have been associated with a weighty increase in the incidence rate that were consistent with some studies [25–27]. This highlights the association between the population environment and the spread. However, in some phases of the pandemic, there have been instances of negative correlation between urbanization and population density, and incidence rate, which has been attributed to the implementation of stay-at-home orders by the government for controlling of the disease. These findings demonstrated the complexity of the relationship between these factors and transmission of COVID-19 and the importance of considering multiple factors in developing strategies to control the spread.

This study suggests that an inverse relationship between hospital density and incidence rate were found out, that means as hospital density increases, incidence rate decreases. However, there is a positive association between the number of medical establishments and incidence rate, meaning that as the number of beds increases, so does the incidence rate. This may be due to the fact that even though there were high density of hospitals, the medical facilities may not have enough resources to effectively address the needs of the population [28].

It is surprising that industry density has been negatively correlated with the incidence rate in some waves of the pandemic. Despite being dense and crowded areas, industries were required to follow strict government rule and regulation to prevent and control the spread, which might have played a role in mitigating transmission in neighboring areas [29, 30]. This highlights the importance of effective realization of public health measures in controlling spread, even for dense and crowded environments.

Significantly, high nighttime light index has been positively associated with incidence rate, indicating that areas with high nighttime light index may have a higher risk in spread. It might be because these areas often have high economic activity and are crowded, creating conditions that facilitate the spread of the virus through aerosol transmission. These findings highlight the importance of considering the relationship between the socioeconomic and the transmission in developing strategies [8].

This study emphasizes the significance of considering the temporality of the factors that drive the transmission of diseases. It highlights that the impact of these factors is not constant but rather changes over time, and it is crucial to monitor and reassess their influence on the spread continuously. This study also underscores the importance of adopting dynamic approaches to public health interventions, as the relationships between different factors and the spread are not fixed but subject to change. The study highlights the need for more in-depth analysis of long-term data to gain a better supportive of the progress of the pandemic and the impact on different interventions on its spread. The findings regarding the association between household density and the virus are particularly relevant, as they demonstrate the need to consider the multifaceted back-and-forth between various factors and the spread of the virus.

These are important limitations of the study that should be considered. The study’s reliance on data availability may have restricted the scope of the analysis and excluded some important factors that could have impacted the spread of COVID-19. The study’s findings should therefore be interpreted with caution, and future research should aim to address these limitations by considering a wider range of timely updated factors (such as percentage of vaccination, health care personal, air pollution) and incorporating more accurate and fine-scale data to better understand the risk factors for COVID-19 transmission. And this study’s used of Spatial Lag and Error Models, while effective, could be complemented by alternative spatial modeling approaches in future research. Methods such as Spatial Autoregressive (SAR) models might offer additional insights into the complex spatial patterns of COVID-19 transmission and the influence of various factors across regions and time periods.

## 5. Conclusion

In Thailand, the pattern of the COVID-19 pandemic shifted over time, as evidenced by the clustering of cases in waves 3 to 5. The clustering of cases and the variation in the impact of factors over different waves highlight the need for adaptive dynamic strategies that consider both spatial and temporal changes. The incidence rate was found to be strongly associated with household density, and the nighttime light index. These factors likely contributed to transmission rates in densely populated and economically active areas. The effects of hospital density and industry density varied over time. The observed inverse relationship between industry density and incidence rate is likely attributable to government containment measures, such as work stoppages, work from home, which reduced economic activity and, consequently, the spread of the virus. The study also found that hospital density can be a protective factor in some phases of the pandemic, but it may become insufficient during times of rapid spread and high numbers of infected individuals. The findings highlight the ongoing challenges in controlling the spread of diseases and the significance of considering multiple factors in developing effective strategies to control the pandemic. Overall, this study emphasizes the importance of implementing and enforcing public health measures to mitigate the transmission of the virus, especially in dense and crowded areas, and in times of high demand for medical resources.

## Supporting information

S1 File Dataset. (XLSX)
